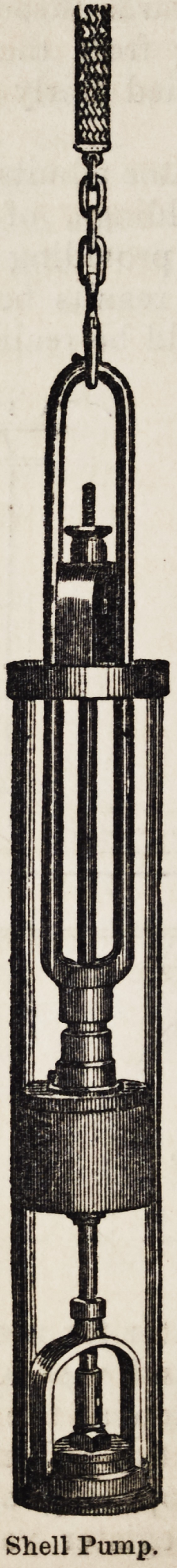# Improvements and Inventions

**Published:** 1855-06

**Authors:** 


### IMPROVEMENTS AND INVENTIONS.

### THE AIR SYPHON VENTILATOR.

We have recently had the opportunity of seeing some experiments on general ventilation with what is called the air-syphon ventilator. The idea of this process originated some years ago with Dr. Chowne.

Fig. 1.

The general principle of the air-syphon ventilator is based on the curious and novel fact that if a tube of the syphon shape (fig. 1) is placed in a room with its long end (C) uppermost, a current of air will immediately play through it in the downward direction of the short (A) and in the upward direction of the long leg (C) of the tube.

The principle of ventilation here involved has been pretty freely canvassed on various occasions, and was, we confess, opposed to our own pre-conceived views. Having, however, carefully observed the experiments upon which this system is based and its practical results, we can speak most favourably concerning it.

The diagram on next page (fig. 2) represents one of the practical illustrations which we have inspected. It represents, in the form of a pillar, the short branch of a syphon communicating with the long branch, by being carried through an orifice in the chimney-board (to which it is fitted), the chimney being in the condition commonly spoken of as a "cold chimney."

Three brackets, each with an argand burner for gas, were fixed on the side of the tube or pillar, and were so arranged that when the chimney-glass of the burner was in its place, its upper edge, on the side next the tube, was on a level with the lowest edge of the short projecting openings or channels, and in contact with it, as represented. Under this arrangement, the demand existing in the chimney for air was dependent for a supply upon the tube, by its open top, and by its lateral short tubular projections above. The room was supplied with air by the usual accessible crevices and openings about doors, windows, &c., and also by other apertures, equivalent to about five inches square, communicating with the open air. Under this arrangement, on smoke paper being applied either to the top of the tube, which was open, or to the small lateral openings in contact with the chimney glasses, the existence of a current which carried the smoke down the tube, was manifest. The gas of the burners was then lighted, and smoke paper again applied, both to the orifice at the top of the tube, and to the projecting openings at the edges of the chimney glasses. The pre-existing current continued, and carried the smoke down the short branch of the tube. The tube, at the same time, necessarily acquired caloric from the heated air which entered it.

The upper orifice of the tube was then covered, to cut off the supply of air, except through the projecting lateral orifices. The air thus passing over the gas flames, acquired very great increase of heat. Prior to the gas being lighted, the temperature within the short branch was 50° F.; in about twenty minutes it rose to 140° F. Thus, although the application of artificial heat was to the short branch, it neither reversed or checked the pre-existing current. On the contrary, with the increased heat of the short branch, the current of air entered with greater velocity over the flames and into the tube.

Fig. 2.

Air, from the extreme mobility of its particles, is a powerful conveyor of heat, and the air passing over the flames of burning gas, and descending in the short branch and rising in the long, carries rapidly with it the heat it acquires; and thus, whatever might be the quantity or bulk of warm air, which the short branch has the capacity for containing, successive repetitions of that bulk or quantity pass from the short to the long branch. The quantity of warm air in the latter thus becomes the multiple of that in the short, by as many times as the cubic contents of the one exceed those of the other: the relative *temperatures* still remaining as before; that is to say, the air in the long branch being relatively cold, and that in the short relatively hot.

It has been considered that the heat emanating from the gas-lights and from persons present, while the doors and windows are closed, would, owing to the elastic qualities of air, cause it to expand, and by such expansion to force a descent into the tube, and so escape. Experience, however, has shewn us that when the windows have been fully opened, so as to put the interior of the room in absolute freedom of communication with the open air, the descent of the hot air through the tube has been uninterrupted : it is, indeed, only while there *is a free admission of air* to the room that the process can be vigorously maintained.

From these experiments we may deduce—

1. That the air of the upper part of a room may be made to pass through an inverted syphon, the descending or short branch of which consists merely of a tube having its opening at the upper part of the room, and communicating with the lower part of the chimney, the ascending or long branch consisting of the chimney itself.

2. That through such a syphon, air is continually passing downwards through the short branch, and upwards through the long.

3. That the deleterious gases, arising from the combustion of coal-gas, may by this contrivance be carried fairly away as fast as they are formed.

The principle of the air-syphon ventilator admits of wide application to the general ventilation of buildings, of ships, and of mines; and if a little care were taken in providing for its application in architectural designs, many useful results both in regard to artistic display and hygienic comfort would be realised.

Figure 3.

Figure 3 is a plan of a portable ventilator for use in sick rooms, nurseries, or other places. The pillar and vase A are placed on the chimney-piece, having a communication at the lowest part by means of a metallic flexible tube B with the chimney. The flexible tube descends and passes in at the opening of the fireplace, and up through the aperture in the register valve C into the chimney.

### EARTH-BORING MACHINERY.

A most ingenious earth-boring machine has recently been brought under notice at the Society of Arts by Messrs. Mather and Platt of Salford. The machine is a great improvement on that of M. Vignoles.

Boring Head.

Shell Pump.

The boring head consists of a wrought-iron bar, about eight feet long, on the lower part of which is fitted a block of cast iron, in which the chisels or cutters are firmly secured. Above the chisels an iron casting is fixed to the bar, by which the boring-head is kept steady and perpendicular in the hole. A mechanical arrangement is provided, by which the boring-head is compelled to move round a part of a revolution at each stroke. The loop or link by which the boring apparatus is attached to the rope is secured to a loose casting on the wrought-iron bar, with liberty to move up and down about six inches. A part of this casting is of square section, but twisted about one-fourth of the circumference. This twisted part moves through a socket of corresponding form on the upper part of a box, in which is placed a series of ratchets and catches, by which the rotary motion is produced.

The shell-pump is a cylinder of cast-iron, to the top of which is attached a wrought-iron guide. The cylinder is fitted with a bucket similar to that of a common lifting pump, with an India-rubber valve.

*Sanitary uses.*—Many small towns, which are so situated that they cannot command a supply of water from natural sources, are prevented from obtaining it by boring, on account of the great expense, and still more the vexatious uncertainty of the process. It is confidently believed that by the new method of boring an abundant supply of water could be procured on the spot, at half that price. Thus in a sanitary point of view, the new machine is of the utmost value; since it enables us to procure a much greater supply of water in far less time, and from depths which were all but inaccessible on the old method of boring.

In conclusion, another important use may be noticed to which this invention may be applied; namely, the ventilation of mines, with a view of preventing the dreadful explosions which are unhappily too frequent. These explosions most frequently arise from the ignition of the gases or foul air accumulated in the galleries, or old workings, and in large cavities which have been partitioned off. The remedy in these cases would be to bore down from the surface and perforate these parts of the mine at different places, so as to admit a current of fresh air into the parts where the foul air had accumulated.

---

### MORTON'S SANITARY PAINT.

We have received some paint from Mr. Morton, of 156, Strand, under the name of "*sanitary paint.*" The process of its manufacture is patented. The advantages which are alleged to be possessed by the paint in a sanitary sense are; that in all colours it is innoxious, and inodorous; that soaps will not remove or injure it; that no oils or turpentine are required to thin it; that a chamber may be slept in on the same night that it is painted; that all pernicious ingredients called dryers are dispensed with; and that the paint is cleanly to use.

A specimen of this paint, forwarded to the laboratory of the Grosvenor-place School of Medicine, and examined by Mr. Rodgers, was found to be a clay paint, and, therefore, like paints of this kind, innoxious: it was possessed of considerable "body."

The main advantage of Morton's paint seems, however, to be that the use of turpentine is dispensed with, whey and soap being used as the vehicle. Deleterious vapours arising from newly painted work are thus prevented. But when poisonous pigments are employed, the workmen must pay the same strict regard to cleanliness as in the use of ordinary paints.

---

### ON THE PURIFICATION OF WATER.

By W. L. Scott, Esq.

In the *Builder*, a few weeks since, there appeared a paragraph headed "Home-made Chloride of Lime," in which Professor Nash recommended the mixing of one barrel of quick-lime with one bushel of salt, the last being previously dissolved in a small quantity of water. This gave, said the writer, a sort of impure chloride of lime, but a powerful deodoriser, equally good for all out-door purposes with the article bought under that name at the apothecary's, and costing not one-twentieth part as much. The incorrectness of this statement being pretty evident, after having tried the mixture, I wrote to the editor of the *Builder* demonstrating that, as proposed, it possesses no disinfecting properties; that it is of less value for purifying than quick-lime alone, as no free chlorine can be evolved from the mixture, and that its components suffer no change whether mixed or otherwise. My letter was inserted, and some weeks have passed without a response from Professor Nash. As the subject materially concerns the public health, I have thought that these facts might perhaps be acceptable, the paragraph in question having been copied into several periodicals, thereby misleading the public, as I think, to some considerable extent.

I enclose the proportion of the mixtures I generally use for disinfecting purposes, and for economising the commercial chloride of lime. The mixture (No. 2) serves for softening and purifying water, results the importance of which, as conducive to public health, will be easily recognised.

1. For disinfecting purposes and economizing chloride of lime:

| | | |
|---|---|---|
| Commercial chloride of lime . . | 4 | lbs. |
| „ peroxide of manganese . | 1 | „ |
| Powdered charcoal . . . | 5 | „ |
| | 10 | „ |

2. For softening and purifying water:

| | | |
|---|---|---|
| Powdered dry oxalate of ammonia . . | 40 | grains |
| „ peroxide of manganese . . | 90 | „ |
| „ charcoal . . . | 350 | „ |
| | 480 = 1 oz. | |

1 oz. of this latter mixture should be agitated with one imperial gallon of the water to be purified, and the clear liquid decanted at the expiration of two or three hours; after which the water, if even permanently hard, as it is called, will be found entirely free from lime and organic matter, thus precluding the necessity of filtering.

The employment of the black oxide of manganese used in the above composition was suggested to me by the fact of my having some time since prevented the putrefaction of small quantities of sea water by its means. The quantity of oxalate of ammonia given above is the exact amount required for a water containing 18·07 grs. of lime per imperial gallon, which is rather above the average of the lime contained in the Thames water. The precise weight of oxalate demanded by any particular water may, of course, be easily ascertained by analysis.

The sanitary and economical considerations of this process being self-evident, I need not dilate upon them, but will merely remark that the results of its use for the last year in several families have proved its efficacy.

[At the suggestion of Mr. Scott, who is a most industrious chemical inquirer, we have for some time past used and recommended the oxalate of ammonia for softening the water used for ablution, and with complete success. The chief danger we see in using Mr. Scott's compound for *drinking* water is, that the oxalate of ammonia, unless very carefully made, may contain a little free oxalic acid. The compound might, therefore, under accidental circumstances lead to deleterious results. We agree with Mr. Scott as to the incorrectness of the suggestion of Professor Nash.—*Editor.*]

## Figures and Tables

**Fig. 1. f1:**
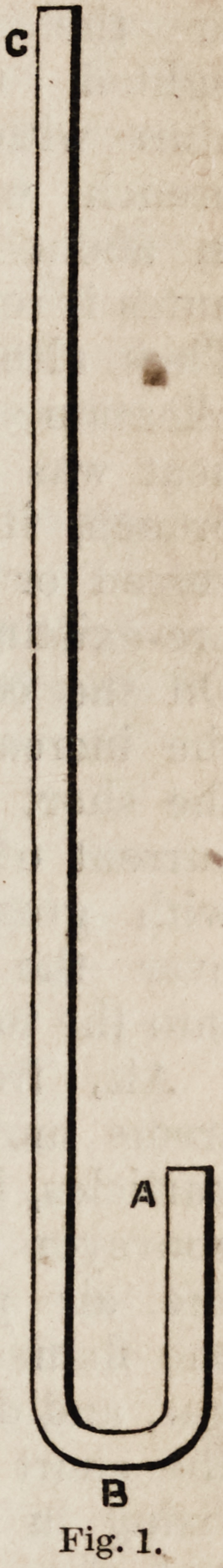


**Fig. 2. f2:**
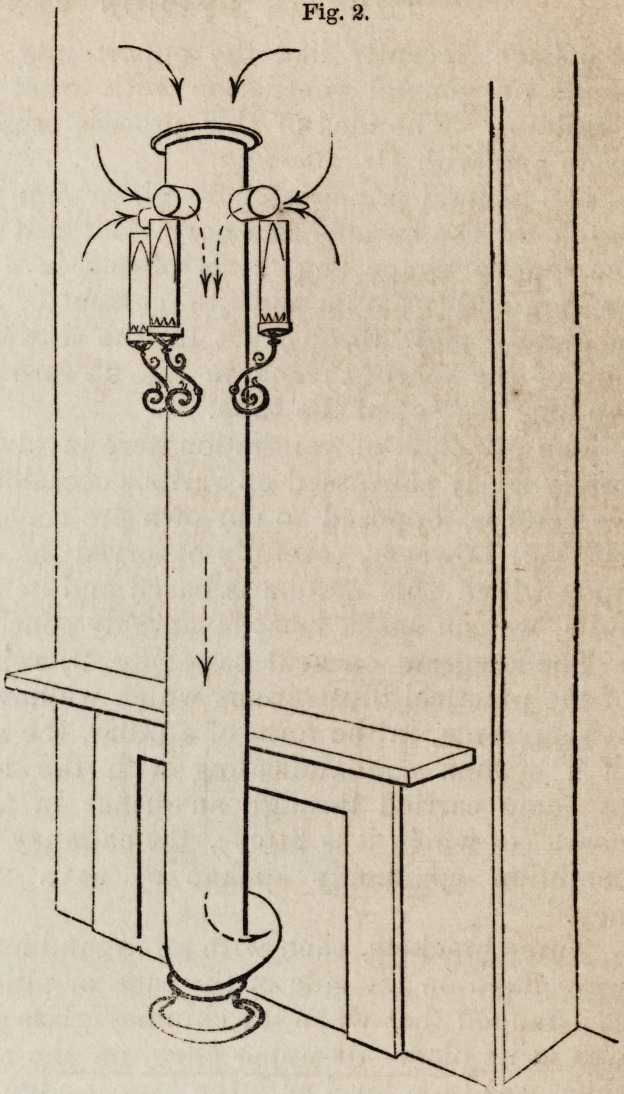


**Figure 3. f3:**
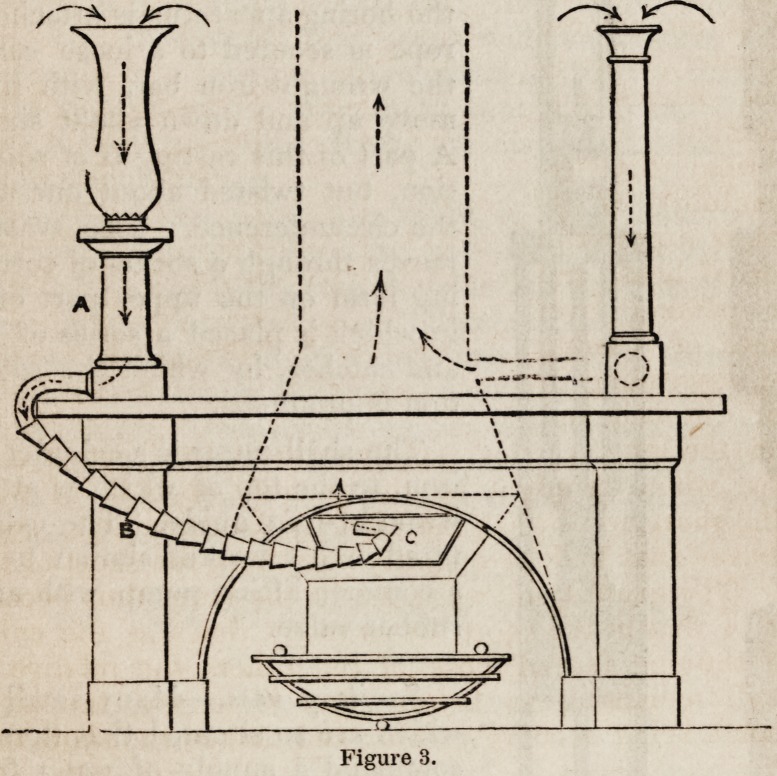


**Figure f4:**
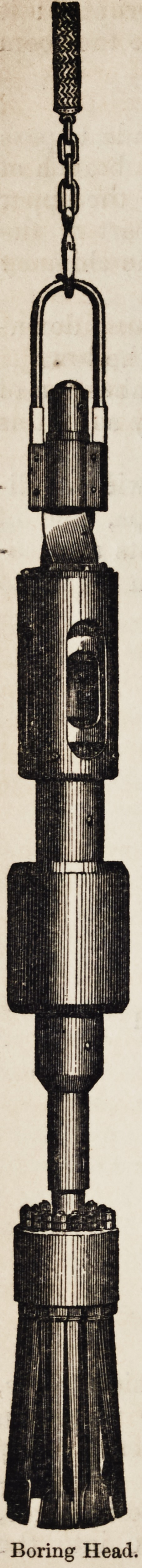


**Figure f5:**